# The Phase Inversion Mechanism of the pH-Sensitive Reversible Invert Emulsion

**DOI:** 10.3390/molecules28217407

**Published:** 2023-11-03

**Authors:** Fei Liu, Yongfei Li, Xiaqing Li, Xuewu Wang

**Affiliations:** 1 College of Petroleum Engineering, Shandong Institute of Petroleum and Chemical Technology, Dongying 257061, China; 2016001@sdipct.edu.cn; 2 Chemistry and Chemical Engineering, Xi’an Shiyou University, Xi’an 710065, China; yfli@xsyu.edu.cn; 3 Petroleum Engineering Technology Research Institute of Shengli Oilfield Company, China Petrochemical Corporation, Dongying 257061, China; lixiaqing.slyt@sinopec.com

**Keywords:** pH-responsive, phase inversion, microstructure, bentonite clay

## Abstract

Reversible emulsification drilling fluids can achieve conversion between oil-based drilling fluids and water-based drilling fluids at different stages of drilling and completion, combining the advantages of both to achieve the desired drilling and completion effects. The foundation of reversible emulsion drilling fluids lies in reversible emulsions, and the core of a reversible emulsion is the reversible emulsifier. In this study, we prepared a reversible emulsifier, DMOB(*N*,*N*-dimethyl-*N*′-oleic acid-1,4-butanediamine), and investigated the reversible phase inversion process of reversible emulsions, including the changes in the reversible emulsifier (HLB) and its distribution at the oil–water interface (zeta potential). From the perspective of the acid–alkali response mechanism of reversible emulsifiers, we explored the reversible phase inversion mechanism of reversible emulsions and reversible emulsification drilling fluids. It was revealed that the reversible phase inversion of emulsions could be achieved by adjusting the pH of the emulsion system. Then the proportion of ionic surfactants changed in the oil–water interface and subsequently raised/lowered the HLB value of the composite emulsifier at the oil–water interface, leading to reversible phase inversion of the emulsion. The introduction of organic clays into reversible emulsification drilling fluid can affect the reversible conversion performance of the drilling fluids at the oil–water interface. Thus, we also investigated the influence of organic clays on reversible emulsions. It was demonstrated that a dosage of organic clay of ≤2.50 g/100 mL could maintain the reversible phase inversion performance of reversible emulsions. By analyzing the microstructure of the emulsion and the complex oil–water interface, we revealed the mechanism of the influence of organic clay on the reversible emulsion. Organic clay distributed at the oil–water interface not only formed a complex emulsifier with surfactants, but also affected the microstructure of the emulsion, resulting in a difficult acid-induced phase transition, an easy alkali-induced phase transition, and improved overall stability.

## 1. Introduction

With the development of oilfields in our country, there has been an increasing demand for higher precision in exploitation and stricter environmental protection requirements. Single working fluids are no longer able to meet the needs of oilfields. The development of working fluids with various properties at different application stages is extremely urgent. An emulsion is a multiphase dispersion system [1], consisting of one liquid dispersed in the form of tiny droplets in another liquid with which it is immiscible [2]. Reversible emulsions can undergo reversible conversion between water-in-oil (O/W) and oil-in-water (W/O) by changing the external conditions, allowing for transitions between different types of emulsions [3,4,5]. Different types of emulsions exhibit distinct properties, such as wetting ability and rheological characteristics, thereby achieving optimal performance [6]. Currently, the main factors affecting reversible emulsions include the pH [7,8], temperature, salinity, pH–temperature synergy, and light.

Reversible invert emulsion fluids are currently primarily used in the field of drilling [9,10]. During the drilling process, oil-based drilling fluids exhibit excellent thermal stability, lubricity, collapse inhibition, and protection of the reservoir. Their performance is significantly superior to water-based drilling fluids. However, during the completion process, the application of oil-based drilling fluids presents several challenges, such as the difficult filter cake removal, the weak bonding strength between cement and the formation, and the handling of oil-contaminated cuttings and waste mud. Therefore, water-based drilling fluids are more effective than oil-based for subsequent completion operations [9,11]. By controlling the acidity and alkalinity of the system, drilling fluids can be converted between oil-based and water-based at different stages of drilling and completion [12], combining the advantages of both to achieve the ideal drilling and completion effects [13,14,15]. The first application of reversible invert emulsion drilling fluids was in 1999 in the Central Graben area of the North Sea, where it was used to assist in grinding operations, demonstrating the advantages of the reversible system in drilling performance and handling cuttings [16]. Reversible invert emulsion drilling fluids have also been used in over 17 wells with more than 30 horizontal laterals in West Africa, with each lateral averaging 3500 ft, totaling more than 100,000 ft [9,17]. In the Cabinda area of Angola, the injection capacity of two wells increased threefold. Furthermore, it has also been experimentally applied in a region of the Gulf of Mexico with formations containing H_2_S and CO_2_, representing a successful attempt at drilling in acid gas-bearing formations using the reversible system [18,19].

Currently, research on reversible invert emulsion drilling fluids has mainly focused on the construction of a reversible invert emulsion drilling fluid system [20,21] and the achievement of phase inversion between the emulsion and the drilling fluid [22,23]. Luyster et al. proposed the combined use of organic acid systems and reversible emulsions, where the organic acid system served as both a chelating agent and a pH regulator to control the inversion of the reversible emulsion [18]. Patel et al. modified surfactants from a molecular structural perspective to give them reversible emulsifying properties [9]. Li et al. focused on the construction of the reversible invert emulsion drilling fluid and developed a system with a density of 1.5 g/cm^3^ and temperature resistance up to 150 °C [20]. However, the mechanism of phase inversion for reversible invert emulsion drilling fluids is not yet thoroughly understood. The authors have previously studied the microstructural changes in the pH-sensitive reversible invert emulsion from W/O to O/W [24]. On this basis, considering that the basis of reversible inversion emulsion drilling fluids is the reversible emulsions, and the core of the reversible emulsions is the reversible emulsifiers, this research revealed the phase inversion mechanism of reversible emulsions and reversible invert emulsion drilling fluids from the perspective of the acid–alkali response mechanism of reversible emulsifiers. The introduction of organic clays into the reversible invert emulsion of a drilling fluid system was studied to examine its effect on the phase inversion performance of reversible invert emulsion drilling fluids, as solid particles of organic clays can be distributed at the oil–water interface [25,26]. However, no specific studies on the effect of organic clay on reversible emulsions have been reported. Therefore, investigating the impact of organic clays on the phase inversion of reversible emulsions will provide a reference for studying the influence of organic clays on reversible invert emulsion drilling fluids, clarify the effect of organic clays on reversible emulsions, and guide the preparation of high-performance reversible invert emulsion drilling fluids.

## 2. Results and Discussion

### 2.1. Characterization of Reversible Emulsion Phase Inversion Performance

**Figure 1:**
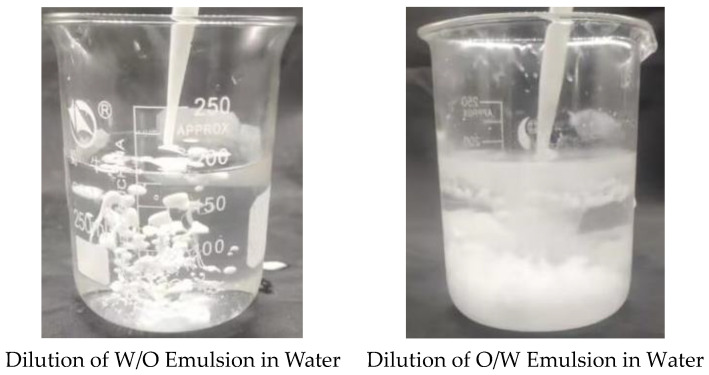
Dilution of the reversible emulsion in water.

The properties of the W/O and O/W emulsion obtained before and after phase inversion of the homemade reversible emulsifier, DMOB, were characterized using the dilution method. The initial W/O emulsion (conductivity: 0 Μs·cm^−1^) was the emulsion before phase inversion, while the O/W emulsion (conductivity: 1002 μS·cm^−1^) was prepared under the condition of 5.00 wt% hydrochloric acid with a dosage of 0.60 vol%. The results of characterization are shown in Figure 1.

### 2.2. Study on the Acid/Alkali Response Mechanism of Reversible Emulsifiers

#### 2.2.1. Response of the Electrical Properties of Oil–Water Interface to Acid/Alkali

The response of the DMOB emulsifier to acid/alkali caused a change in the proportion of ion-type surfactants at the oil–water interface, which, in turn, affected the electrical charge (zeta potential) of the emulsion droplets. Therefore, the changes in the zeta potential during the phase inversion process of the reversible emulsion were investigated. Due to the poor conductivity of the oil phase in the W/O emulsion, it was challenging to measure the zeta potential. Thus, the zeta potential of the emulsions at different stages of the O/W emulsion phase inversion process was tested to analyze the changes in the electrical properties of the oil–water interface during this process.

The generation of an electrical charge (zeta potential) in the emulsion droplets is due to the presence of ion-type surfactants at the oil–water interface. In this case, the variations in zeta potential mainly resulted from changes in the proportion of ion-type surfactants at the oil–water interface (with the total amount of surfactants remaining constant and the overall size of the oil droplets in the O/W emulsion being relatively consistent). This analysis aimed to examine the impact of acid/alkali on the electrical properties of the oil–water interface in reversible emulsions.

According to the effect of the dosage of acid/alkali on the zeta potential of W/O emulsion, the surface electrification of the W/O emulsion increased with an increase in the dosage of hydrochloric acid and decreased with an increase in the dosage of the sodium hydroxide solution. The main reason is that the proportion of the oil–water interface of the ionic surfactant increased with an increase in the dosage of hydrochloric acid, and the proportion of the oil–water interface of the ionic surfactant decreased with an increase in the dosage of the sodium hydroxide solution.

**Figure 2:**
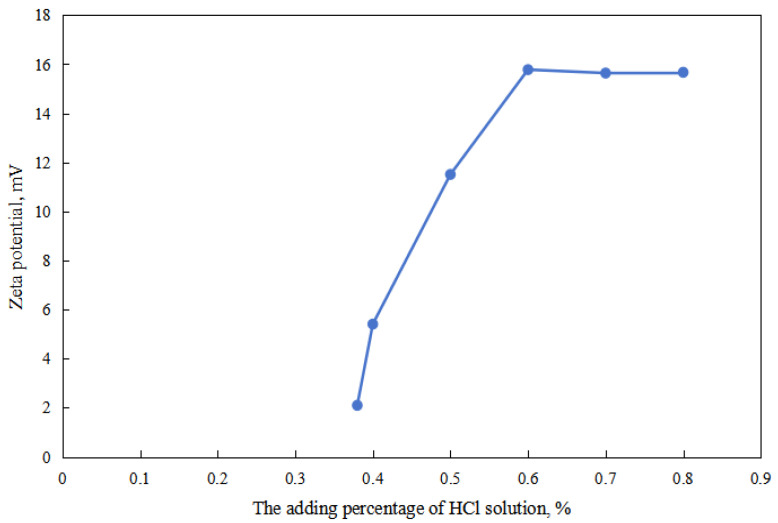
The effect of the acid on the zeta potential of the O/W emulsion.

**Figure 3:**
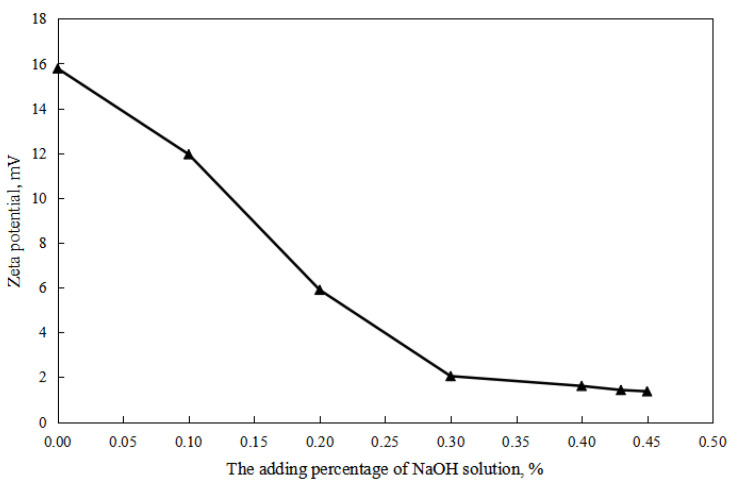
The effect of alkali on the zeta potential of the O/W emulsion.

**Figure 4:**
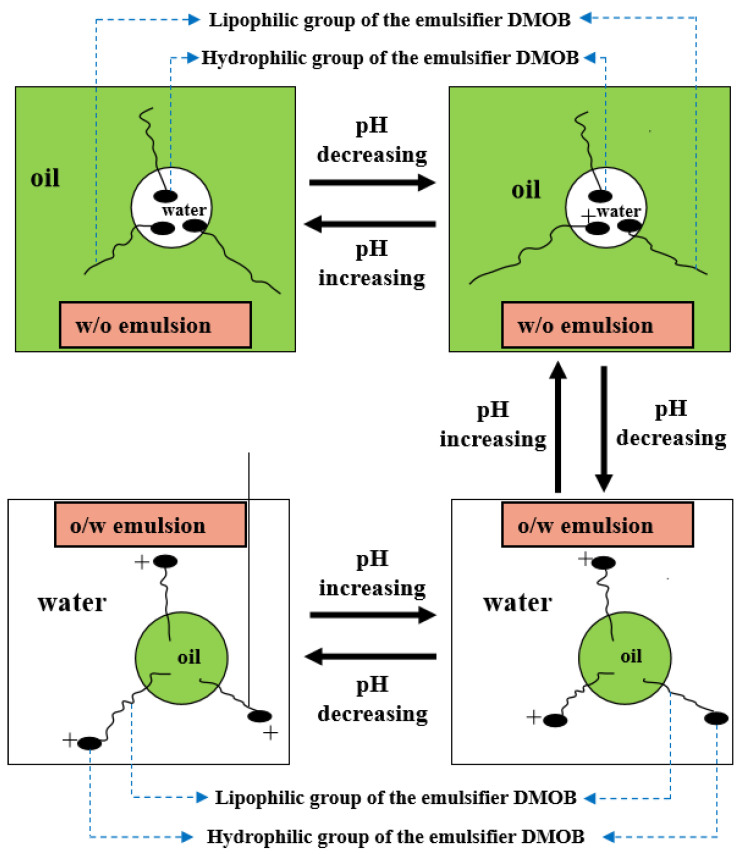
The effect of acid/alkali on the composition of the emulsifier at the oil–water interface of the emulsion.

As shown in Figure 2 and Figure 3, firstly, when the reversible emulsion underwent an acid-induced phase inversion, the zeta potential of the resulting O/W emulsion was 2.10 mV, which was higher than the minimum value of 1.37 mV achieved during the alkali-induced phase inversion of the O/W stage. This result indicated that the acid-induced phase inversion of the reversible emulsion could only occur when the zeta potential reached a relatively higher value. In other words, the reversible emulsion could not undergo acid-induced phase inversion unless there was a significant conversion of non-ionic surfactants to ionic surfactants at the oil–water interface. It is only when the proportion of ion-type surfactants at the oil–water interface reached a certain threshold that acid-induced phase inversion could be achieved in the reversible emulsion. Secondly, the zeta potential of the reversible emulsion decreased with an increase in the amount of the sodium hydroxide solution in the W/O emulsion stage during the alkali-induced phase inversion process of the reversible emulsion. Prior to the alkali-induced phase inversion of the reversible emulsion, the zeta potential did not reach a fixed value. The phase conversion point test of the reversible emulsion indicated that the amount of the sodium hydroxide solution (5.00 wt%) required for the reversible emulsion to achieve the alkaline conversion phase (0.45 vol%) was much smaller than the amount of hydrochloric acid (5.00 wt%) required for the preparation of the initial oil-in-water emulsion (0.6 vol%). The results indicated that the alkaline conversion phase of the emulsion was completed before the ionic surfactant at the oil–water interface had completely transformed into a non-ionic surfactant (Figure 4). The fundamental reason for the effect of acid/alkali on the electric properties of the oil–water interface of reversible emulsions is the acid/alkali response of the reversible DMOB emulsifier.

#### 2.2.2. Response of the Emulsifier’s HLB Value to Acid/Alkali

The HLB value of the emulsifier is an index used to characterize the type of emulsion that the emulsifier can stabilize. Therefore, the influence of the amount of acid/alkali on the HLB value of the reversible emulsifier was tested experimentally, and the influence of the amount of acid/alkali on the hydrophilic and oleophilic properties of the emulsifier at the oil–water interface was analyzed. The amount of DMOB emulsifier in the reversible emulsion system was 2.5 g/ 200 mL, so the amount of acid/alkali in the DMOB emulsifier corresponding to the reversible emulsion system (Z vol%) was Z mL/1.25 g.

##### HLB Values Required for Emulsifying Cottonseed Oil and Kerosene into O/W Emulsions

(1) Through experiments, it was determined that the ratio of oleic acid to sodium oleate, which could emulsify cottonseed oil and deionized water into a water-in-oil emulsion and had the best stability was 23:11. The HLB value of this composite emulsifier (6.5) was the HLB value required for the emulsification of cottonseed oil into a water-in-oil emulsion, that is, E was 6.5.
(2) Through experiments, it was determined that the ratio of oleic acid to sodium oleate was 11:23, which could emulsify kerosene and deionized water to form a W/O emulsion and had the best stability. The HLB value of this compound emulsifier (12.5) was the HLB value required for the emulsification of kerosene into a W/O emulsion, and F was 12.5.

##### Influence of Acid/Alkali on the Reversible Emulsifier’s HLB Value

(1) Effect of the acid solution on the HLB value of the reversible emulsifier

In the initial stages with a lower dosage of hydrochloric acid, a composite emulsifier was prepared by combining the DMOB emulsifier with sodium oleate in different ratios. The optimal ratio was able to successfully emulsify kerosene with deionized water into a stable O/W emulsion. The HLB value of this composite emulsifier was found to be 12.5.

**Figure 5:**
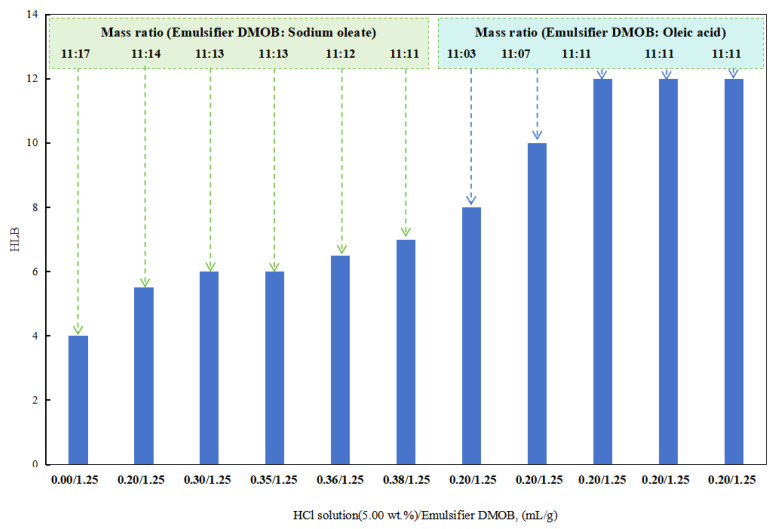
The effect of acid on the HLB of the emulsifier.

After obtaining a higher HLB value for the DMOB emulsifier, the following method was used for testing. The DMOB emulsifier was combined with oleic acid in different ratios to find the optimal ratio for effectively emulsifying cottonseed oil with deionized water into a stable O/W emulsion (Figure 5). The HLB value of this composite emulsifier was determined to be 6.5. Hydrochloric acid at a concentration of 5.00 wt% was used for this purpose.

(2) Influence of alkali on the reversible emulsifier’s HLB value

First, 0.60 mL/1.25 g of hydrochloric acid (5.00 wt%) was added to the DMOB emulsifier and mixed thoroughly. Different amounts of the sodium hydroxide solution (5.00 wt%) were then added to determine the HLB value of the DMOB emulsifier.

In the initial stages with a lower dosage of the sodium hydroxide solution (5.00 wt%), a composite emulsifier was prepared by combining the DMOB emulsifier with oleic acid at different ratios. The optimal ratio was able to successfully emulsify cottonseed oil with deionized water into a stable O/W emulsion. The HLB value of this composite emulsifier was found to be 6.5.

**Figure 6:**
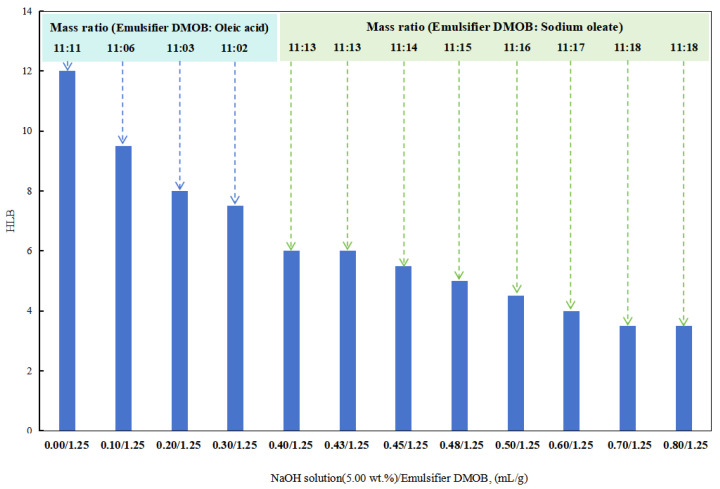
The effect of the NaOH solution on the HLB of the emulsifier.

After obtaining a lower HLB value for the DMOB emulsifier, the following method was used for testing. The DMOB emulsifier was combined with sodium oleate in different ratios to find the optimal ratio for effectively emulsifying kerosene with deionized water into a stable O/W emulsion (Figure 6). The HLB value of this composite emulsifier was determined to be 12.5.

When the dosage of the sodium hydroxide solution (5.00 wt%) in the DMOB emulsifier was 0.40 mL/1.25 g and 0.43 mL/1.25 g, the W/O emulsion could be prepared directly with SKLAN 5# white oil and deionized water, but the phase conversion process was more complicated. In other words, when the amount of sodium hydroxide in the reversible emulsion system was 0.40 vol% and 0.43 vol%, the alkali-induced phase transformation did not occur, but the conductivity of the reversible emulsion was greatly reduced, which was consistent with the results that the emulsifier system in the emulsion system was no longer suitable for stabilizing the oil-in-water emulsion, but it was also unable to complete the phase transformation or break the emulsion. As a result, an unstable water-in-oil emulsion was formed.

The reversible conversion process of the reversible emulsifier is basically the same as the reversible phase process of the corresponding reversible emulsion. The phase transition lag occurs only at the phase transition point due to the complexity of the emulsion’s phase transition. The phase transformation of emulsions can be divided into general phase transformation and dynamic phase transformation. General phase transformation means that the emulsion obtained under certain emulsifying conditions is a W/O emulsion (or an O/W emulsion), and the type of emulsion prepared by slightly changing the emulsifying conditions changes. Dynamic phase transition means that under specific dynamic shear conditions, changing the formula or composition of the existing emulsion will cause the phase transition of the emulsion. Because the dynamic phase transformation of the emulsion needs to destroy the existing emulsion system first, the difficulty of dynamic phase transformation is generally higher than that of ordinary phase transformation. General phase transformation must be realized if dynamic phase transformation can be achieved, while general phase transformation may not necessarily be realized if dynamic phase transformation can be achieved [27,28,29,30]. According to the change in the HLB value of the emulsifier, the type of emulsion stabilized by the emulsifier changes with the change in the amount of acid/alkali, which belongs to the general phase transformation of the emulsion, while the phase transformation of the emulsion achieved by adding acid/alkali to the emulsion system belongs to the dynamic phase transformation, and the difficulty of dynamic phase transformation is generally higher than that of general phase transformation, which is the cause of the lag in the phase transformation of the emulsion.

#### 2.2.3. The Reversible Emulsion’s Multi-Repeat Phase Conversion Performance

The advantages of reversible emulsions not only concentrate the advantages of different types of liquid, but also have high environmental value due to their phase reversibility, which can be repeated many times. Therefore, the repeated phase transfer performance of reversible emulsions is very important for their application. The number of reversible emulsions determines the number of times they can be reused, but the repeated reversibility of reversible emulsions leads to higher requirements for the shear resistance of reversible emulsifiers and the range of oil–water ratios adapted to the system. The reversible emulsion prepared by DMOB was tested for its multiple repeated reversible phase performance, and the reversible emulsifier was completely converted into an ionic emulsifier at a dosage of 0.60 vol% of hydrochloric acid (5.00 wt%). The alkaline dosage was a sodium hydroxide solution with the same number of moles as the H+ contained in hydrochloric acid.

**Figure 7:**
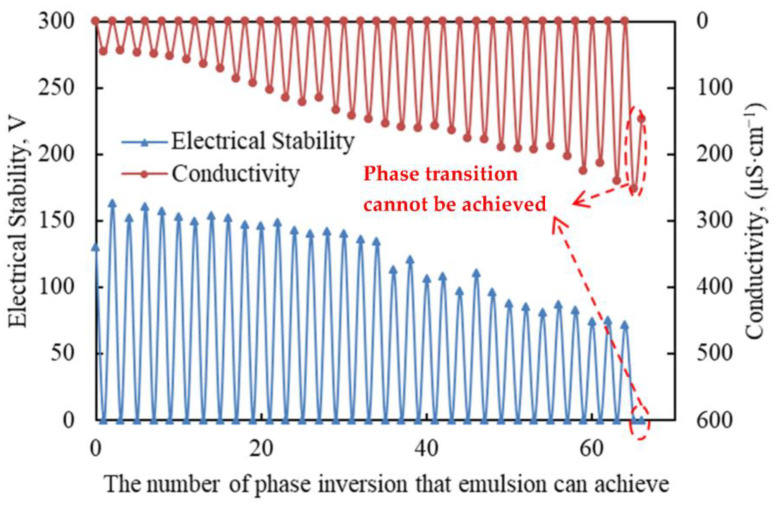
The properties of multiple reversible phase inversions.

The experimental results are shown in Figure 7, indicating that the reversible emulsion could achieve 65 phase transformations, which is better than the reversible emulsion liquid system of Shengli Oilfield, which has 10 sets of phase transformations. The results show that the DMOB emulsifier had good shear resistance and the reversible emulsion system could adapt to a wide range of oil–water ratios and had good repetitive phase transformation performance.

### 2.3. Study on the Influencing Mechanism of Organic Clay on Reversible Emulsion

In light of the current main use of reversible emulsion, organic clay is required to be added to the reversible emulsion drilling fluid system to adjust the rheological properties and stability of the reversible emulsion drilling fluid [31,32], so as to improve the cutting–carrying performance of the reversible emulsion drilling fluid [33]. Therefore, studying the influence of organic clay on reversible emulsions is beneficial for the promotion and application of homemade reversible emulsion systems in oil fields.

Therefore, it is necessary to study the reversible phase properties of emulsions prepared under different dosages of organic clay and to determine the range of dosages of organic clay that can prepare reversible emulsions (dosage of organic clay ≤ 2.50 g/100 mL) (Table 1).

To determine the dosage range of organic clay, 0.00 g/100 mL, 1.00 g/100 mL, 2.00 g/100 mL, and 2.50 g/100 mL dosages of organic clay were selected. The study investigated the changes in the properties of the emulsion (static stability, demulsification voltage, conductivity, and microstructure) with increasing dosages of hydrochloric acid under different dosages of organic clay, and analyzed the impact of organic clay on reversible emulsions.

Since the response factor of a reversible emulsion’s phase conversion is acid/alkali, the study of the effect of organic clay on the reversible emulsion’s phase conversion should first investigate whether organic clay will consume acid/alkali and whether the organic clay will consume H^+^/OH^−^. The organic clay dispersion system involved thoroughly stirring the organic clay in the liquid system (organic clay was added while stirring. Then the mixture was stirred for 5 min at 300 r/min. The container was sealed during the mixing process) before the tests were conducted.

**Figure 8:**
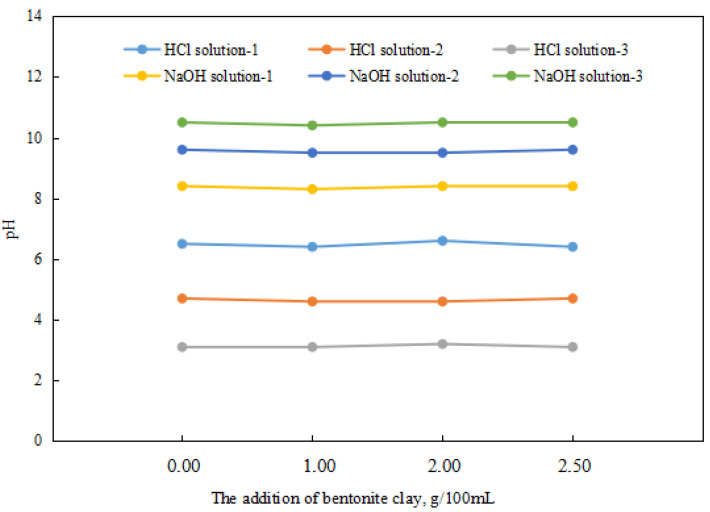
The pH of the dispersion system with different added amounts of organic clay.

Different amounts of organic clay had almost no effect on the pH values of the acid and alkali systems (Figure 8). The organic clay used in the experiment did not consume H^+^ and OH^−^ in acid and alkali environments. Moreover, according to the structural analysis of organic clay, it did not consume H^+^ and OH^−^, so it was not necessary to consider the consumption of H^+^ and OH^−^ by organic clay when studying the influence of organic clay on the reversible emulsion’s phase transition.

#### 2.3.1. Influence of Organic Clay on Acid-Induced Phase Transition

**Figure 9:**
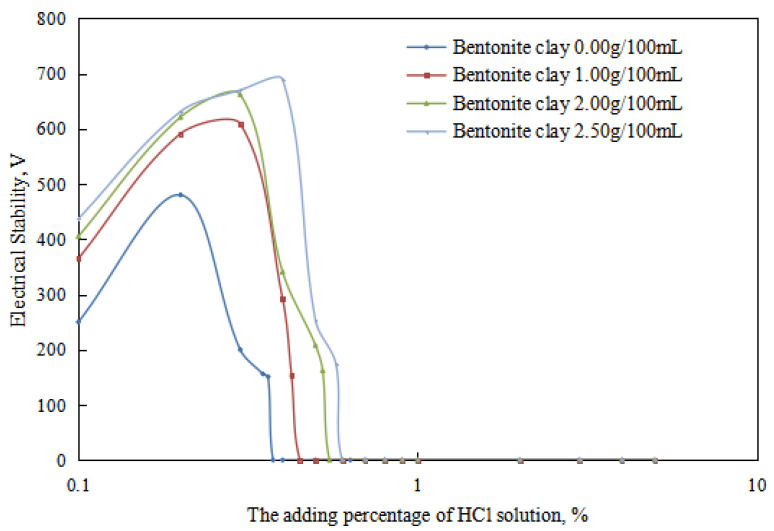
The effect of hydrochloric acid on the emulsion-breaking voltage of the emulsion.

**Figure 10:**
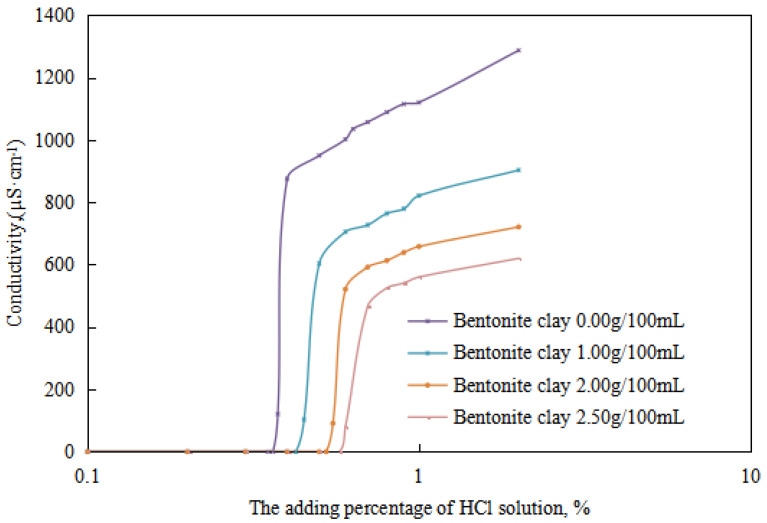
The effect of hydrochloric acid on the conductivity of the emulsion.

The variations in the demulsification voltage and the conductivity of the emulsion with different dosages of organic clay and acid were studied (Figure 9 and Figure 10). A hydrochloric acid concentration of 5.00 wt% was used to analyze the changes in the acid-induced phase transition point of the reversible emulsion with increasing dosages of organic clay.

Under different organic clay dosages, the acid conversion phase point of the reversible emulsion (Figure 9 and Figure 10) was as follows: (1) 0.38 vol% hydrochloric acid with 0.00 g/100 mL organic clay, (2) 0.45 vol% hydrochloric acid with 1.00 g /100 mL organic clay, (3) 0.55 vol% hydrochloric acid with 2.00 g/100 mL organic clay, and (4) 0.60 vol% hydrochloric acid with 2.50 g/100 mL organic clay. Therefore, a further comprehensive analysis was carried out, combined with the microstructure of the emulsion.

Cryo-TEM testing and analysis were performed on the initial W/O emulsion of the reversible emulsion under different dosages of organic clay. With the addition of organic clay to the emulsion system, a film of organic clay and the surfactant of the composite emulsifier formed at the oil–water interface [34,35].

**Figure 11:**
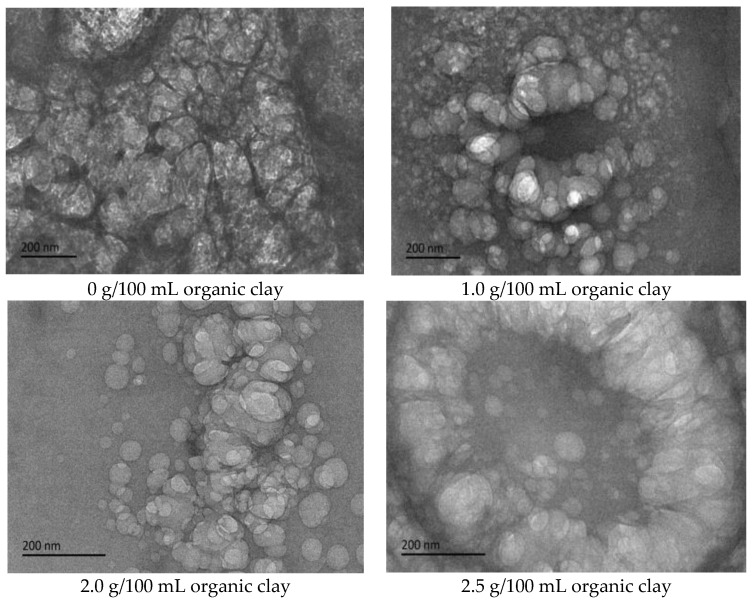
The effect of organic clay on the micromorphology of the W/O emulsion (cryo-TEM).

The proportion of organic clay in the composite emulsifier film increased with an increase in the amount of organic clay, and the organic clay was lipophilic. Therefore, the composite emulsifier film exhibited stronger lipophilicity with an increase in the proportion of organic clay, resulting in an increase in the acid conversion phase point of the reversible emulsion with an increase in the amount of organic clay, that is, the addition of organic clay made it more difficult for the reversible emulsion to achieve the acid conversion phase. As can be observed in Figure 11, the compactness between water droplets in the W/O emulsion increased with an increase in the dosage of organic clay, resulting in the enhanced stability of the emulsion droplets and making it more difficult for the emulsion to undergo the acid-induced phase transition. Moreover, the tight structure between the water droplets hindered the free movement of hydrogen ions, further increasing the difficulty of the acid-induced phase transition in the emulsion.

**Figure 12:**
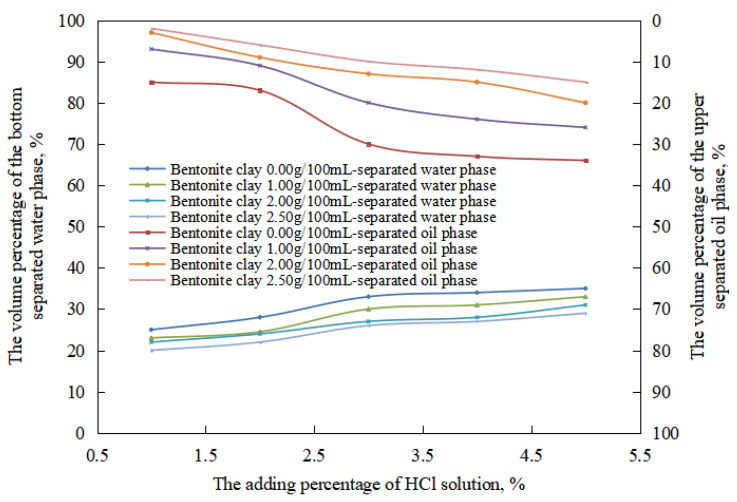
The effect of hydrochloric acid on the standing stability of an O/W emulsion with organic clay.

Due to the small number of stages of the oil-in-water emulsion in the reversible acid-induced phase transformation process, which included the equal-conversion stage of the oil-in-water emulsion, the changes in the stability of the oil-in-water emulsion with the amount of organic clay in this process were compared (Figure 12). In the oil-in-water emulsion stage, under the same pH value, the water extraction rate and the oil extraction rate of the emulsion after standing for 24 h decreased with an increase in the amount of organic clay. This is because the organic clay formed a composite emulsifier film with the surfactant at the oil–water interface. With an increase in the dosage of organic clay, the proportion of organic clay in the composite emulsifier film increased, enhancing the stability of the composite emulsifier film and, consequently, the stability of the O/W emulsion increased with an increasing dosage of organic clay. Therefore, with an increase in the amount of organic clay, the difficulty of destroying the oil droplets increased, and the oil extraction rate decreased. At the same time, the amount of destruction of the oil drops and precipitation decreased with an increase in the amount of organic clay within 24 h. Therefore, after 24 h, the volume of the oil phase in the oil-in-water emulsion increased with an increase in the amount of organic clay, and the water evolution rate decreased.

#### 2.3.2. Influence of Organic Clay on Base-Induced Phase Transition

**Figure 13:**
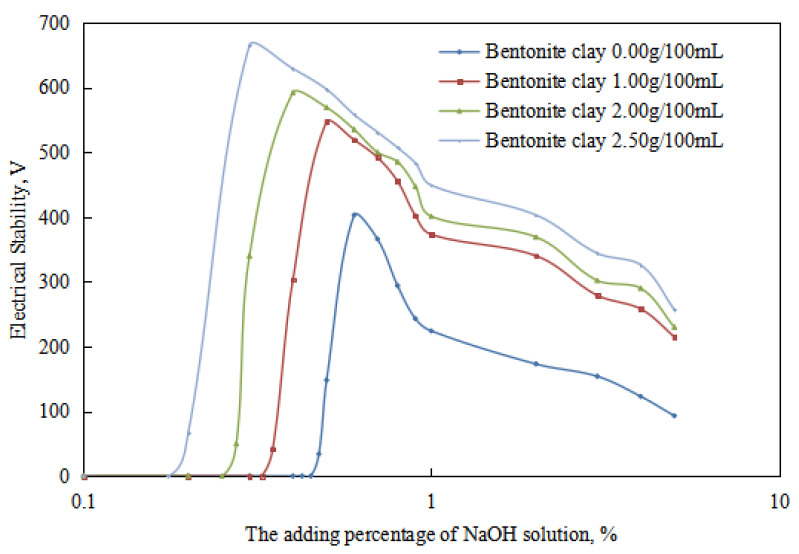
The effect of the NaOH solution on the emulsion-breaking voltage of the emulsion.

**Figure 14:**
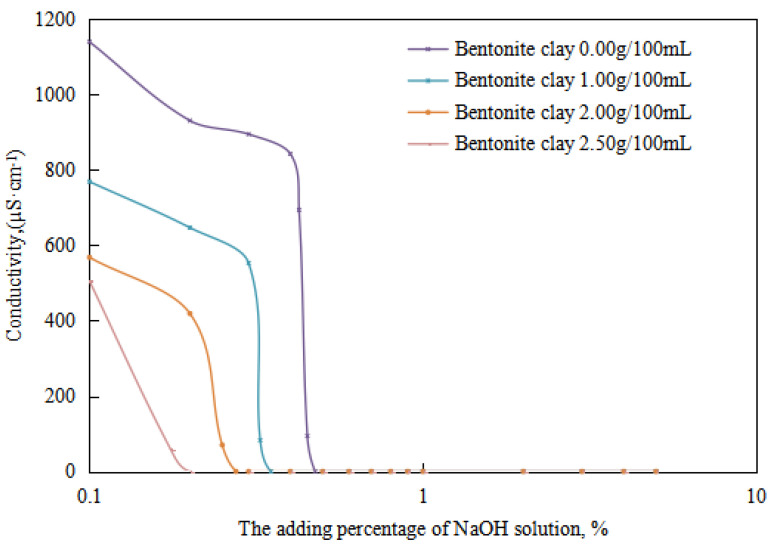
The effect of the NaOH solution on the conductivity of the emulsion.

The changes in the emulsion’s demulsification voltage and electrical conductivity with an increase in the dosage of the sodium hydroxide solution were studied with different dosages of organic clay (Figure 13 and Figure 14). On this basis, the changes in the alkali-induced transition phase point of the reversible emulsion with an increase in the dosage of organic clay were analyzed. The alkaline modifier used was a sodium hydroxide solution with a concentration of 5.00 wt%.

The alkali-induced phase points of the reversible emulsions with different amounts of organic clay can be obtained from the trend diagram above (the initial O/W emulsion had a pH of 6.0): (1) a sodium hydroxide solution dosage of 0.48 vol% with an organic clay dosage of 0.00 g/100 mL, (2) a sodium hydroxide solution dosage of 0.35 vol% with an organic clay dosage of 1.00 g/100 mL, (3) a sodium hydroxide solution dosage of 0.28 vol% with a organic clay dosage of 2.00 g/100 mL and (4) a sodium hydroxide solution dosage of 0.20 vol% with a organic clay dosage of 2.50 g/100 mL. A further comprehensive analysis was conducted in conjunction with the microstructure of the emulsion.

**Figure 15:**
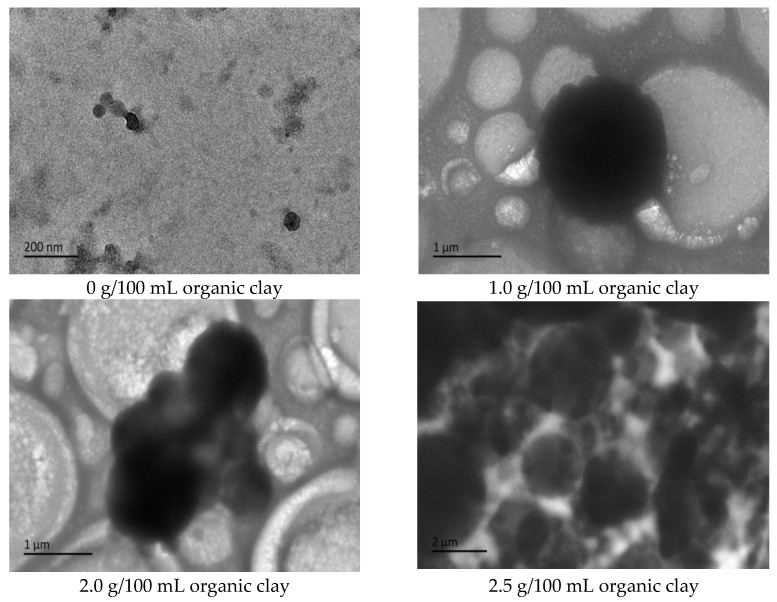
The effect of organic clay on the micromorphology of initial O/W emulsion (cryo-TEM).

To further analyze the influence of the dosage of organic clay on the alkali-induced phase transition of the reversible emulsion, cryo-TEM testing was performed on the initial O/W emulsion of the reversible emulsion under different dosages of organic clay. With the addition of organic clay to the emulsion system, a composite emulsifier film consisting of organic clay and the surfactant formed at the oil–water interface. The proportion of organic clay in the composite emulsifier film increased with an increase in the dosage of organic clay. The organic clay exhibited hydrophobic properties, resulting in the stronger hydrophobic nature of the composite emulsifier film with a higher proportion of organic clay. This led to a decrease in the alkali-induced phase transition point of the reversible emulsion with an increase in the dosage of organic clay, indicating that the addition of organic clay made it easier for the reversible emulsion to undergo the alkali-induced phase transition. As shown in Figure 15, the overall particle size of the oil droplets in the W/O emulsion increased with an increase in the amount of organic clay, resulting in a decrease in the amount of lye required for the reversible emulsion’s alkaline phase transition, and a decrease in the difficulty of the reversible emulsion’s alkaline phase transition.

**Figure 16:**
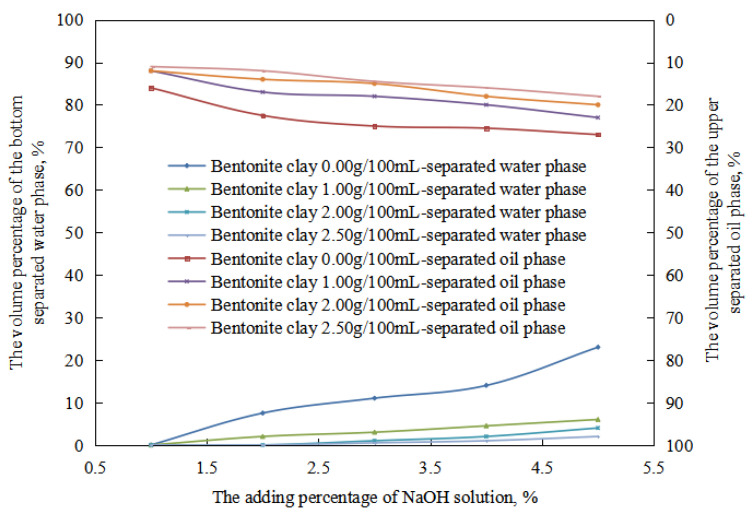
The effect of the NaOH solution on the standing stability of the W/O emulsion.

In the process of the alkaline conversion of reversible emulsions, there are few stages of oil-in-water emulsions, and there are equal phases of oil-in-water emulsions. Therefore, the stability of the oil-in-water emulsion with different amounts of organic clay was mainly compared in this process (Figure 16). At the stage of a water-in-oil emulsion, under the same pH value, the water-in-oil emulsion’s water-out rate and oil-out rate decreased with an increase in the amount of organic clay after standing for 24 h. The main reason was that a composite emulsifier film of the organic clay and the surfactant formed when organic clay was added to the liquid emulsion system. The proportion of organic clay in the composite emulsifier film increased with an increase in the amount of organic clay, and the stability of the composite emulsifier film increased with an increase in the proportion of organic clay. Thus, the stability of the water-in-oil emulsion was enhanced with an increase in the amount of organic clay, and the tightness of the water droplets’ structure was enhanced with an increase in the amount of organic clay. The structure between the water droplets was more difficult to destroy with an increase in the amount of organic clay. The water droplets were difficult to pack tightly, so the oil extraction rate of the W/O emulsion decreased with an increase in the amount of organic clay.

In summary, the organic clay and surfactant formed a composite emulsifier film at the oil–water interface, and the proportion of organic clay in the composite emulsifier film increased with an increase in the dosage of organic clay. Because the stability of the composite emulsifier film was higher than that of the simple surfactant film, both the stability of the O/W emulsion and the W/O emulsion increased with an increase in the dosage of organic clay. At the same time, because the organic clay was lipophilic, and the tightness of the water droplets of the oil-in-water emulsion was enhanced with an increase in the amount of organic clay, the amount of acid required for the reversible emulsion’s acid phase transformation increased with an increase in the amount of organic clay. Because the organic clay was oleophilic, and the overall particle size of the droplets of W/O emulsion increased with the increased amounts of organic clay, and the uniformity of particle size became worse with an increase in the amount of organic clay. Therefore, the difficulty of the alkaline phase transformation of the reversible emulsion decreased with an increase in the amount of organic clay, and the amount of alkali required for the phase transformation of the reversible emulsion decreased with an increase in the amount of organic clay.

## 3. Materials and Methods

### 3.1. Materials

**Figure 17:**
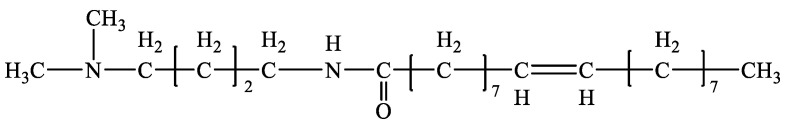
The molecular structure of the DMOB.

The main reagents used in the experiment were purchased from China National Pharmaceutical Group Chemical Reagent Co., Ltd. (Shanghai, China), including hydrochloric acid, sodium hydroxide, oleic acid, and sodium oleate. The organic clay NTC (industrial grade) was provided by Shengli Oilfield Drilling Engineering Technology Co., Ltd. (Dongying, China) The Skylan No. 5 white oil was purchased from Skylan Petroleum (Chongqing) Co., Ltd. (Chongqing, China) The DMOB emulsifier was made in our laboratory (Figure 17). Cottonseed oil and kerosene (industrial grade) were purchased from Shunda Oil Products Co., Ltd. (Shandong) (Zibo, China).

### 3.2. Experimental Apparatus

We used a DWY-2A intelligent electrical stability Tester (Qingdao Xinling Electromechanical Technology Co., Ltd., Qingdao, China), an NGJ-2 Mud High-Speed Mixer (Qingdao Jiaonan Analytical Instrument Factory, Qingdao, China) a conductivity meter (DDS-307, Shanghai Jingke, Shanghai, China), an FA1004 electronic balance (Shanghai Fangrui Instrument Co., Ltd., Shanghai, China), an XSP-11CE transmitted/reflected biological microscope (Shanghai Changfang Optical Instrument Co., Ltd., Shanghai, China), a pH meter (PHSJ-3F, Shanghai Jingke, Shanghai, China), a Brookhaven zeta potential and particle size analyzer (Brookhaven Instruments Corporation, Holtsville, NY, USA) and a JEM-1400 Plus transmission electron microscope (JEOL Ltd., Tokyo, Japan).

### 3.3. Experimental Method

#### 3.3.1. Determination of the HLB Value for Reversible Emulsifiers

(1) Calculation of the HLB value of composite emulsifier

(1) $$HLB=\frac{AC}{A+B}+\frac{BD}{A+B}$$

We mixed Emulsifier 1 (Ag) with 2 (Bg), where the HLB value of Emulsifier 1 was *C* and the HLB value of Emulsifier 2 was *D*. The HLB value of the composite emulsifier was calculated as shown in Equation (1).

(2) Preparation of the emulsion

We added 5 parts of the tested composite surfactant to 15 parts of the oil phase, then added 80 parts of water. To emulsify, a high-speed mud mixer was used at 3000 rpm for 5 min. The HLB value of the surfactant in the sample with optimal stability in the emulsion was considered as the required HLB value of the oil phase.

(3) HLB value for emulsification of cottonseed oil and kerosene into an O/W emulsion

The HLB values of oleic acid and the sodium oleate were 1 and 18.

We mixed oleic acid and sodium oleate in different proportions to determine the ratio that could form a stable O/W emulsion of cottonseed oil and deionized water. The HLB value of this composite emulsifier was considered as the required HLB value for emulsifying cottonseed oil into an O/W emulsion and was temporarily designated as E [36].

We mixed oleic acid and sodium oleate in different proportions to determine the ratio that could form a stable O/W emulsion of kerosene and deionized water. The HLB value of this composite emulsifier was considered as the required HLB value for emulsifying kerosene into an O/W emulsion and was temporarily designated as F [36].

(4) HLB value of emulsifiers with different dosages of acid/alkali

Surfactants with HLB values of 3–6 can stabilize W/O emulsions, while surfactants with HLB values of 7–18 can stabilize O/W emulsions.

In the stage of the low dosage of hydrochloric acid, we could mix the surfactant with sodium oleate in different proportions to find the ratio that could emulsify kerosene and deionized water into the O/W emulsion with the optimal stability. The HLB value of this composite emulsifier was F.

When HLB value of a surfactant was greater than 7, we used the following method for testing. We mixed the surfactant with oleic acid in different proportions and found the ratio that could emulsify cottonseed oil and deionized water into the O/W emulsion with the optimal stability. The HLB value of this composite emulsifier was E.

#### 3.3.2. Preparation of the Initial Reversible Emulsion

We mixed 2.5 g of the DMOB emulsifier and 100 mL of Skylan No. 5 white oil. Then we added 100 mL of deionized water. The mixture was stirred at a speed of 12,000 r/min for 10 min to form the initial W/O emulsion. This was labelled as the Type I emulsion.

#### 3.3.3. Study on the Phase Inversion Performance of Reversible Emulsions

To test the acid-induced phase inversion, we conducted parallel experiments with different groups. For this, 100 mL of the Type I emulsion was taken for each group, and we added varying volumes of hydrochloric acid with a concentration of 5.00 wt% (0–5 vol%) to the emulsions. The mixture was stirred at 12,000 r/min for 5 min. We tested the emulsions from each group for the demulsification voltage, conductivity, stability upon standing, pH value, and the dispersed droplets’ morphology. The O/W emulsion prepared with 0.6 vol% of hydrochloric acid was labelled as the Type II emulsion.

To test the alkali-induced phase inversion (acid-induced phase inversion), we conducted parallel experiments with different groups. For this, 100 mL of the Type II emulsion was taken for each group, and we added varying volumes of the sodium hydroxide solution with a concentration of 5.00 wt% (0–5 vol%) to the emulsions. The mixture was stirred at 12,000 r/min for 5 min. We tested the emulsions from each group for the demulsification voltage, conductivity, stability upon standing, pH value, the dispersed droplets’ morphology, viscosity, rheology, and particle size distribution.

The stability of the W/O emulsion was characterized by the demulsification voltage, while the continuity of the water phase in the O/W emulsion was characterized by the conductivity of the emulsion. The static stability of reversible emulsions was studied by changing the amount of acid/alkali under different conditions, and the stability in each stage of the reversible phase process was also characterized.

#### 3.3.4. Study on the Stability of Reversible Emulsions

We let the emulsion stand and observed the separation of oil and water, and recorded the volume of the oil phase and the water phase separated from the emulsion over time. The time when the water phase or oil phase obviously precipitated was called the emulsion’s static stabilization time. We calculated the separation rate of water and oil according to the volume of water and oil in the emulsion [37]. The separation rates mentioned in this article are the rates of change in water and oil of the emulsion after standing for 24 h.

(2) $$Water\;separation\;rate=\frac{Volume\;of\;separated\;water\;phase}{Total\;volume\;of\;emulsion}$$

(3) $$Oil\;separation\;rate=\frac{Volume\;of\;separated\;oil\;phase}{Total\;volume\;of\;emulsion}$$

## 4. Conclusions

In summary, this research studied the phase-changing process of a reversible emulsion. By reducing/increasing the pH value of the emulsion system, the proportion of ionic surfactants in the oil–water interface film could be increased/reduced, so as to increase/reduce the HLB value of the oil–water interface of the composite emulsifier, and finally achieve a phase change in the reversible emulsion. At the same time, according to the experimental results of the reversible emulsifier, DMOB, the range of dosages of organic clay with reversible phase changes was determined (≤2.50 g/100 mL). The effect of organic clay on the properties of the reversible emulsion was further studied, and its mechanism was analyzed from the perspective of the microstructure of the emulsion and the composite oil–water interface of the film. The results showed that the composite emulsifier film was formed at the oil–water interface between the organic clay and the surfactant, and the stability of the emulsion was enhanced with an increase in the amount of organic clay. In addition, the organic clay was lipophilic, and with an increase in the amount of organic clay, the tightness of the water droplets in the W/O emulsion increased, and the amount of acid required for the reversible emulsion’s acid response increased. Correspondingly, the droplet size of the O/W emulsion increased with an increase in the amount of organic clay, and the uniformity of particle size decreases with an increase in the amount of organic clay, and the amount of lye required for the reversible emulsion’s alkaline response decreased.

## Figures and Tables

**Table 1:** The effect of organic clay on the reversible phase properties of emulsions.

Dosage of Organic Clay (g/100 mL)	Feasibility of Preparing the Initial O/W Emulsion	Feasibility of Acid-Induced Phase Transition	Feasibility of Alkali-Induced Phase Transition
0.00	Yes	Yes	Yes
1.00	Yes	Yes	Yes
2.00	Yes	Yes	Yes
2.50	Yes	Yes	Yes
3.00	Yes	No	-

## Data Availability

The data presented in this study are available wholly within the manuscript.
